# Assessment of hormonal levels as prognostic markers and of their optimal cut-offs in small intestinal neuroendocrine tumours grade 2

**DOI:** 10.1007/s12020-020-02534-8

**Published:** 2020-11-26

**Authors:** Dimitrios Papantoniou, Malin Grönberg, Kalle Landerholm, Staffan Welin, Barbara Ziolkowska, Dennis Nordvall, Eva Tiensuu Janson

**Affiliations:** 1 Department of Medical Sciences, Endocrine Oncology, Uppsala University, Uppsala, Sweden; 2 Department of Oncology, Ryhov County Hospital, Jönköping, Sweden; 3 Department of Surgery, Ryhov County Hospital, Jönköping, Sweden; 4 Maria Sklodowska-Curie National Research Institute of Oncology, Gliwice, Poland; 5 Qulturum, Region Jönköping, Jönköping, Sweden

**Keywords:** Chromogranin A, 5HIAA, Small intestinal neuroendocrine tumours, Biomarker, Carcinoids, Cut-offs

## Abstract

**Purpose:**

Small intestinal neuroendocrine tumours (siNETs) with a Ki-67 proliferation index between 3 and 20% belong to WHO grade 2. Response to treatment may be monitored by blood chromogranin A (CgA) and urine 5-hydroxyindoleacetic acid (5HIAA). The aim of this retrospective study was to investigate the prognostic value of baseline CgA and 5HIAA and of the early biochemical response to treatment, and to compare different cut-off values used in the literature.

**Methods:**

A retrospective cohort study of 184 patients with siNET Grade 2 treated with somatostatin analogues (SSA), interferon-alpha (IFN) or peptide receptor radionuclide therapy (PRRT).

**Results:**

Baseline CgA was a statistically significant prognostic marker for both cancer-specific survival (CSS) and progression-free survival (PFS). A cut-off of 5 × ULN (upper limit of normal) was best discriminative in most cases, but 2 × ULN discriminated better for SSA. Baseline 5HIAA was a prognostic marker for CSS in treatment with IFN and PRRT, but not for single SSA. Early changes of CgA and 5HIAA correlated well with CSS (HR 3.18, 95% CI 1.82–5.56 and HR 1.47, 95% CI 1.16–1.86) and PFS (HR 3.08, 95% CI 1.86–5.10 and HR 1.37, 95% CI 1.11–1.68) for SSA, but not for PRRT.

**Conclusions:**

Baseline CgA and to a lesser extent 5HIAA are associated with CSS irrespective of treatment used, and with PFS after PRRT, and 5 × ULN provides best discrimination in many, but not all, cases. Early reductions of CgA and 5HIAA are prognostic for treatment with SSA, but not PRRT.

## Introduction

Neuroendocrine neoplasms (NENs) of the small intestine are the third largest subgroup of NENs in the gastroenteropancreatic system [1]. According to the WHO classification from 2019, they are grouped on the basis of their proliferation index into well differentiated grade 1 (G1, Ki-67 < 3%), grade 2 (G2, Ki-67 3–20%) and grade 3 (G3, Ki-67 > 20%) neuroendocrine tumours (NETs) and the rare, poorly differentiated G3 neuroendocrine carcinomas (NEC, Ki-67 > 20%) [2]. In a recent Surveillance Epidemiology and Ends Results database analysis, which uses an older slightly different classification system, well differentiated NETs were approximately four times more common than intermediate grade tumours [3]. NETs G2 have a more aggressive clinical behaviour than their G1 counterparts and probably altered molecular background [4].

Two monoanalytes, plasma chromogranin A (CgA) and urine 5-hydroxyindoleacetic acid (5HIAA) are used to monitor the course of these neoplasms during treatment of non-curable tumours, and may also have a role in detection of recurrence after potentially curative surgery or other treatments [1]. CgA has been studied for two decades as a potential diagnostic marker for NENs. However, more recent work has failed to support this idea, and a recent Delphi consensus concluded that no single biomarker meets the sensitivity and specificity standards to be considered as a diagnostic tool [5, 6]. Although conflicting evidence exists regarding their prognostic and predictive value, most studies report that at least higher CgA levels at baseline are associated with shorter survival, while significance of CgA changes for predicting worse response to treatment is still unclear. [7–9]. When dichotomizing these biomarkers, different authors have been using various cut-offs ranging from 1 to 10 times the upper limit of normal (×ULN), leading to results that are not entirely comparable [7, 10–16]. Limited data shows that early reduction of CgA and/or 5HIAA may correlate with treatment effect of some, but not all anti-tumoural agents, and with the survival of the patient [17, 18].

Despite differences between G1 and G2 small intestinal NETs (siNETs), most studies to date have examined them as a uniform group, and often in combination with NETs of other origin. Their results are thus more representative of the much more common G1 tumours. To the best of our knowledge, this is the first study investigating the association between baseline CgA and 5HIAA, as well as their change during treatment, in a uniform cohort of patients with G2 tumours of the small intestine, treated with somatostatin analogues (SSA), peptide receptor radionuclide therapy (PRRT) and/or interferon-alpha (IFN).

## Materials and methods

In this retrospective cohort study, all patients with metastatic siNETs with a Ki-67 proliferation index between 3 and 20% (WHO grade 2) that were treated at the Department of Endocrine Oncology, Uppsala University Hospital, a tertiary referral centre, and at the Department of Oncology, Ryhov County Hospital, a regional hospital, between 1 January 2000 and 31 May 2017 were eligible for inclusion and were retrieved from an internal database. Patients treated by surgery after initiation of cancer-specific treatment, with no evidence of remaining disease and no recurrence during the study period, were excluded from the survival analysis. Following approval from the Uppsala ethical review board (Dnr 2017/403), data on patients’ clinical status, treatments given and laboratory tests were extracted from the hospitals’ medical archives. Medical records were re-reviewed to determine cause of death and survival status was censored on 31st October 2019 or at last known contact. Causes of death due to tumour progression, adverse events, surgical morbidity as well as cases where cause of death was indeterminate but cancer-related death was likely, were classified as cancer-specific mortality. Patients dying from other causes, not related to their NET tumour, were censored at time of death. Cancer-specific survival (CSS) and progression-free survival (PFS) were calculated for each treatment given. Progression was defined as radiological progression, according to multidisciplinary team assessment, whenever available. It is worth noting that RECIST criteria were not consistently applied during the study period, and that in a small subset of mostly older patients (less than 10%) treating physician’s assessment of biochemical/clinical progression was accepted as time for progression.

Biomarkers were collected at baseline, and at the 6-month visit. In a minority of patients undergoing interventional procedures within this 6-month period, last biomarker control before intervention was accepted, as long as it was at least 3 months after treatment start; otherwise these patients were excluded from analysis. For the measurement of 5HIAA, patients were provided with one or two receptacles and were asked to collect urine for one or two 24-h periods prior to the planned visit and to maintain a serotonin-poor diet for 72 h beforehand. 5HIAA was measured as a single sample or as two samples on consecutive days; whenever two samples were examined, the mean value was used. Samples were analyzed using high-performance liquid chromatography. Plasma samples for CgA were collected in chilled heparinised vacutainer tubes after fasting overnight. All samples before and during PRRT were measured at Uppsala University Hospital using the EuroDiagnostica kit (Malmö, Sweden) for CgA. Samples before and during other treatments were measured at Uppsala University Hospital (85% of evaluable samples for patients treated with SSA or IFN) or at the patient’s local laboratory. In each case, baseline and 6-month tests were conducted at the same laboratory. Levels of CgA and 5HIAA were described as ×ULN of the reporting laboratory.

Biochemical partial response (PR) was defined as a reduction of baseline CgA or 5HIAA by at least 50% and biochemically progressive disease (PD) as an increase by at least 25%. Patients with values at 6 months between −50% and +25% of baseline were deemed as having biochemically stable disease (SD).

### Statistical methods

Statistical analysis was performed with R version 3.5.3, and the compareGroups package 4.0.0, using standard methodology (chi-square test for dichotomous variables, *t*-test for continuous variables and semi-parametric cox models for censored variables). PFS and CSS were analyzed using the Kaplan–Meier method and between‐group differences were analyzed using a log‐rank test. Hazard ratios (HRs) and confidence intervals (CIs) were estimated from the Cox proportional hazards model. Optimal cut-off points were calculated with R packages Survminer 0.4.3 and maxstat 0.7–25 using the maximally selected rank statistics, a method that allows the evaluation of cut-off points, which provide the classification of different risk groups in a quantitative or ordered predictor variable [19, 20]. Sensitivity, specificity, positive (PPV) and negative predictive values (NPV) were estimated with R package timeROC 0.3.

All tests were two-sided. *P* values < 0.05 were considered statistically significant.

## Results

### Demographics

**Fig. 1:**
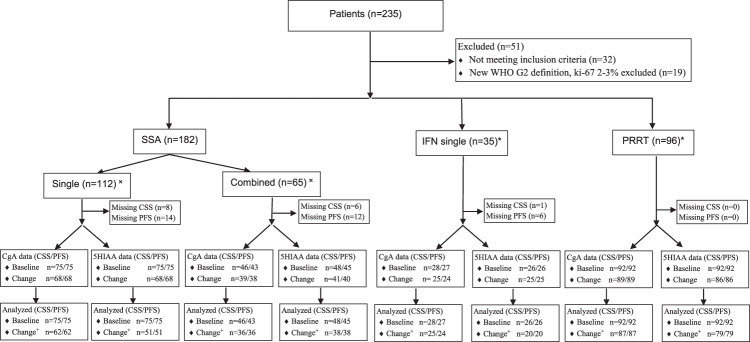
SSA somatostatin analogues, IFN interferon, PRRT peptide receptor radionuclide therapy, CgA Chromogranin A, 5HIAA 5-hydroxyindoleacetic acid, CSS cancer-specific survival, PFS progression-free survival, 5HIAA 5-hydroxyindoleacetic acid. ˟Five patients with uncertain status excluded. *Treatments given. Most patients received several lines of treatment. Four patients were rechallenged with IFN and 13 with PRRT, and were included as separate treatment events. Approximately one third of patients treated with SSA and IFN had missing baseline and/or 6-month CgA and 5HIAA data. ^+^For SSA single, SSA combined, IFN, and PRRT, 6, 3, 0 and 2 cases with baseline normal CgA and 17, 3, 5 and 7 cases with normal baseline 5HIAA were excluded from early biochemical response analysis

In total, 184 patients with metastatic siNET G2 were included in the present study. During the study period 182 patients were treated with SSA, 93 patients with IFN and 92 patients with PRRT. Four patients were rechallenged with IFN and 13 with PRRT, and were included as separate treatment events. Additional treatments including chemotherapy and everolimus were offered to less than 20% of patients and were not analyzed. Most patients started treatment with SSA. PRRT was used mostly as second or third line of treatment. The patterns of use of IFN changed significantly during the study period: During the initial years, it was used either as single first line treatment (13 patients) or in parallel with SSA, often starting at the same time or within a few months of SSA initiation (62% of the IFN treatment cases). During the latter phase of the study IFN was mostly used as second or third line treatment. Baseline biochemical markers and selected demographics are summarized in Table 1. A consort diagram is shown in Fig. 1.

**Table 1:** Baseline patient characteristics

	SSA (*N* = 182)	IFN (*N* = 97)	PRRT (*N* = 105)
Sex			
Female	73 (40.1%)	40 (41.2%)	40 (38.1%)
Male	109 (59.9%)	57 (58.8%)	65 (61.9%)
Age at start (median [IQR])	64.7 [58.3;71.5]	60.9 [53.7;68.2]	65.9 [58.4;71.1]
Resection of primary tumour/lymph nodes			
No	47 (26.4%)	20 (20.8%)	20 (19.2%)
Yes	131 (73.6%)	76 (79.2%)	84 (80.8%)
Metastasectomy			
No	122 (67.0%)	56 (57.7%)	64 (61.0%)
Yes	60 (33.0%)	41 (42.3%)	41 (39.0%)
Performance status (WHO)			
0	65 (58.0%)	34 (58.6%)	52 (54.7%)
1	31 (27.7%)	15 (25.9%)	33 (34.7%)
2	12 (10.7%)	8 (13.8%)	9 (9.5%)
≥3	4 (3.6%)	1 (1.7%)	1 (1.1%)
Ki-67 (median [IQR])	7% [4%;10%]	6% [4%;10%]	8% [5%;12%]
Bone/lung metastases			
No	114 (75.5%)	50 (67.6%)	49 (49.0%)
Yes	37 (24.5%)	24 (32.4%)	51 (51.0%)
Line of treatment			
1	168 (93.3%)	60 (62.5%)	9 (8.7%)
2	12 (6.7%)	29 (30.2%)	60 (57.7%)
≥3	0 (0.0%)	7 (7.3%)	35 (33.7%)
Concomitant start SSA/other			
No	112 (63.3%)	35 (41.7%)	96 (91.4%)
Yes	65 (36.7%)	49 (58.3%)	9 (8.6%)
Baseline CgA, × ULN (median [IQR])	6.8 [2.1;34.0]	8.0 [2.2;45.2]	16.5 [4.5;32.0]
Baseline CgA, dichotomized			
<5	49 (40.5%)	31 (43.7%)	27 (26.7%)
5–10	17 (14.0%)	6 (8.5%)	12 (11.9%)
>10	55 (45.5%)	34 (47.9%)	62 (61.4%)
Baseline 5HIAA, × ULN (median [IQR])	4.6 [1.3;15.5]	4.2 [1.1;15.0]	6.1 [2.7;15.0]
Baseline 5HIAA, dichotomized			
<5	68 (55.3%)	40 (58.0%)	42 (41.6%)
5–10	16 (13.0%)	7 (10.1%)	24 (23.8%)
>10	39 (31.7%)	22 (31.9%)	35 (34.7%)

*SSA* somatostatin analogues, *IFN* Interferon-alpha, *PRRT* peptide receptor radionuclide therapy, *CgA* chromogranin A, *5HIAA* 5-hydroxyindoleacetic acid, *ULN* upper limit of normal, *IQR* interquartile range

### Baseline biomarkers as prognostic markers

We examined the association between biomarker (CgA and 5HIAA) levels at treatment start and survival (CSS and PFS) both as continuous and dichotomous variables. Baseline CgA was consistently a statistically significant prognostic marker for both CSS and PFS as a continuous variable (non-significant association in the case of PFS on SSA) (Table 2). Baseline 5HIAA was a statistically significant prognostic marker for CSS in patients treated with IFN and PRRT, but not in patients treated with single SSA, and a statistically significant prognostic marker of PFS for patients treated with PRRT (Table 3).

**Table 2:** Baseline CgA as predictor for CSS, PFS

	SSA single		SSA combined		IFN		PRRT	
	HR [95% CI]	*p*	HR [95% CI]	*p*	HR [95% CI]	*p*	HR [95% CI]	*p*
CSS								
	*N* = 75		*N* = 46		*N* = 28		*N* = 92	
Continuous	1.008 [1.003;1.012]	**<0.01**	1.008 [1.004;1.011]	**<0.01**	1.012 [1.004;1.020]	**<0.01**	1.016 [1.008;1.023]	**<0.01**
5–10×		**0.02**		0.10		**0.02**		**<0.01**
5–10×	1.39 [0.49;3.97]		0.85 [0.22;3.22]		3.37 [0.56;20.28]		5.90 [1.97;17.66]	
>10	2.78 [1.32;5.87]		2.12 [0.95;4.75]		6.01 [1.56;23.21]		9.06 [3.75;21.88]	
>2	5.30*** [1.85;15.21]	**<0.01**	1.49 [0.61;3.67]	0.38	2.65 [0.58;12.05]	0.19	NA	
>5	2.27 [1.12;4.62]	**0.02**	1.76 [0.80;3.87]	0.15	5.15* [1.40;18.99]	**0.01**	8.44*** [3.51;20.31]	**<0.01**
>10	2.55 [1.29;5.01]	**0.01**	2.21*** [1.06;4.64]	**0.03**	4.30 [1.37;13.51]	**0.01**	4.56 [2.45;8.50]	**<0.01**
>Optimal: treatment	5.30*** [1.85;15.21]	**<0.01**	1.49 [0.61;3.67]	0.38	23.88*** [4.47;127.62]	**<0.01**	8.32 [2.97;23.25]	**<0.01**
>Optimal	2.88 [1.43;5.82]	**<0.01**	1.71 [0.81;3.61]	0.15	5.15 [1.40;18.99]	**0.01**	6.38 [2.94;13.85]	**<0.01**
PFS								
	*N* = 75		*N* = 43		*N* = 27		*N* = 92	
Continuous	1.002 [0.998;1.006]	0.35	1.004 [1.002;1.007]	**<0.01**	1.005 [1.000;1.010]	**0.04**	1.007 [1.003;1.011]	**<0.01**
5–10×		0.29		0.36		**0.03**		**<0.01**
5–10×	1.64 [0.79;3.39]		0.88 [0.27;2.83]		6.78 [1.02;45.11]		3.88 [1.13;13.29]	
>10	1.45 [0.82;2.57]		1.61 [0.74;3.48]		4.62 [1.22;17.55]		6.36 [2.58;15.72]	
>2	2.22*** [1.21;4.07]	**0.01**	0.94 [0.40;2.18]	0.87	4.24 [0.54;33.00]	0.14	4.01 [0.96;16.80]	**0.04**
>5	1.50 [0.89;2.55]	0.13	1.40 [0.67;2.95]	0.38	4.90*** [1.33;17.99]	**0.01**	5.94*** [2.41;14.63]	**<0.01**
>10	1.27 [0.75;2.14]	0.38	1.67* [0.82;3.38]	0.16	3.05 [0.99;9.38]	**0.04**	4.06 [2.01;8.21]	**<0.01**
>Optimal: treatment	2.22*** [1.21;4.07]	**0.01**	2.87*** [1.25;6.63]	0.01	4.90*** [1.33;17.99]	**0.01**	5.43 [2.34;12.59]	**<0.01**
>Optimal	1.64 [0.97;2.76]	0.06	1.72 [0.83;3.60]	0.15	4.90 [1.33;17.99]	**0.01**	5.41 [2.33;12.55]	**<0.01**

Prognostic value of baseline Chromogranin A (CgA) for cancer-specific (CSS) and progression-free survival (PFS) given as continuous values, at “standard” cut-offs 2×, 5×, 10 × ULN and at “optimal” estimated cut-offs for the whole cohort (optimal) and per treatment given (optimal: treatment). Significant *p* values are marked in bold numbers. Hazard ratios providing best discrimination are marked with *** (all cut-offs) and with * for “standard” cut-offs, when different

*ULN* upper limit of normal, *SSA* somatostatin analogues, *IFN* Interferon-alpha, *PRRT* peptide receptor radionuclide therapy, *NA* not available

**Table 3:** Baseline 5HIAA as predictor for CSS, PFS

	SSA single		SSA combined		IFN		PRRT	
	HR [95% CI]	*p*	HR [95% CI]	*p*	HR [95% CI]	*p*	HR [95% CI]	*p*
CSS								
	*N* = 75		*N* = 48		*N* = 26		*N* = 92	
Continuous	1.014 [0.989;1.040]	0.26	1.014 [1.002;1.026]	**0.02**	1.064 [1.013;1.119]	**0.01**	1.071 [1.038;1.104]	**<0.01**
5–10×		0.07		**0.02**		0.17		**0.01**
5–10×	2.50 [0.98;6.36]		0.38 [0.09;1.69]		1.35 [0.27;6.73]		2.08 [1.04;4.15]	
>10	2.03 [0.92;4.45]		2.01 [0.96;4.21]		3.19 [0.90;11.39]		2.60 [1.39;4.85]	
>2	1.62 [0.79;3.31]	0.18	1.37 [0.59;3.18]	0.47	1.08 [0.35;3.33]	0.89	1.49 [0.73;3.06]	0.27
>5	2.18* [1.10;4.34]	**0.02**	1.33 [0.65;2.72]	0.43	2.20 [0.71;6.84]	0.16	2.36* [1.34;4.17]	**<0.01**
>10	1.70 [0.81;3.60]	0.16	2.42*** [1.18;4.94]	**0.01**	2.98* [0.89;9.97]	0.06	1.97 [1.15;3.38]	**0.01**
>Optimal: treatment	2.54*** [1.25;5.18]	**0.01**	1.08 [0.51;2.30]	0.84	3.89*** [1.22;12.40]	**0.01**	6.30*** [3.24;12.27]	**<0.01**
>Optimal	1.98 [1.00;3.95]	**0.05**	1.50 [0.73;3.06]	0.26	2.63 [0.83;8.32]	0.09	2.57 [1.48;4.46]	**<0.01**
PFS								
	*N* = 75		*N* = 45		*N* = 26		*N* = 92	
Continuous	1.003 [0.985;1.022]	0.74	1.001 [0.985;1.017]	0.90	1.026 [0.986;1.067]	0.21	1.048 [1.012;1.087]	**0.01**
5–10x		**0.05**		0.40		0.26		**0.01**
5–10x	2.54 [1.16;5.55]		1.15 [0.41;3.22]		3.03 [0.74;12.45]		3.06 [1.46;6.40]	
>10	1.33 [0.72;2.46]		1.65 [0.79;3.44]		1.88 [0.47;7.61]		1.97 [0.99;3.95]	
>2	1.64* [0.95;2.81]	0.07	1.29 [0.56;2.99]	0.54	1.69 [0.52;5.54]	0.38	1.86 [0.73;4.74]	0.19
>5	1.59 [0.93;2.72]	0.09	1.49 [0.74;2.97]	0.26	2.32* [0.74;7.27]	0.14	2.34*** [1.26;4.36]	**0.01**
>10	1.17 [0.65;2.12]	0.60	1.59*** [0.80;3.15]	0.18	1.46 [0.39;5.46]	0.57	1.30 [0.72;2.34]	0.39
>Optimal: treatment	2.08*** [1.11;3.90]	**0.02**	1.35 [0.63;2.91]	0.43	2.53*** [0.75;8.54]	0.12	2.34*** [1.26;4.36]	**0.01**
>Optimal	1.55 [0.90;2.65]	0.11	1.49 [0.74;2.97]	0.26	1.63 [0.49;5.46]	0.42	2.34*** [1.26;4.36]	**0.01**

Prognostic value of baseline 5-hydroxyindoleacetic acid (5HIAA) for cancer-specific (CSS) and progression-free survival (PFS) given as continuous values, at cut-offs 2×, 5×, 10 × ULN and at “optimal” estimated cut-offs for the whole cohort (optimal) and per treatment given (optimal: treatment). Significant *p* values are marked in bold numbers. Hazard ratios providing best discrimination are marked with *** (all cut-offs) and with * for “standard” cut-offs

*ULN* upper limit of normal, *SSA* somatostatin analogues, *IFN* Interferon-alpha, *PRRT* peptide receptor radionuclide therapy

Using the R package Survminer, we estimated optimal cut-off points for each treatment and for the whole cohort, irrespective of treatment. The estimated optimal cut-offs for different treatments varied considerably (Table 4). For the group as a whole, suggested cut-offs were 6 × ULN of both CgA and 5HIAA for CSS. Respective cut-offs for PFS were 6 × ULN and 5 × ULN.

**Table 4:** Optimal cut-offs (×ULN)

	CgA		5HIAA	
	CSS	PFS	CSS	PFS
Irrespective of treatment	**6**	**6**	**6**	**5**
SSA single	2	2	4	1
SSA combined	90	45	17	6
IFN	32	5	8	3
PRRT	4	7	18	5

Estimated “optimal” cut-offs for Chromogranin A (CgA) and 5-hydroxyindoleacetic acid (5HIAA) using the maximally selected rank statistics, for the cohort as a whole (in bold), irrespective of treatment, and per treatment given

*ULN* upper limit of normal, *SSA* somatostatin analogues, *IFN* Interferon-alpha, *PRRT* peptide receptor radionuclide therapy, *CSS* cancer-specific survival, *PFS* progression-free survival

Subsequently, we calculated HRs for CSS and PFS at “standard” dichotomized cut-off values used in the literature 2×, 5×, 10 × ULN [12, 15, 16, 21] and at a trichotomized cut-off (5–10 × ULN), as well as at the estimated optimal cut-off points for CgA and 5HIAA. Results for each treatment given are summarized in Tables 2 and 3, and for all cases, irrespective of treatment, in Supplementary Table 1. Sensitivity, specificity, PPV and NPV are shown in Supplementary Table 2.

### Somatostatin analogues (SSA)

**Fig. 2:**
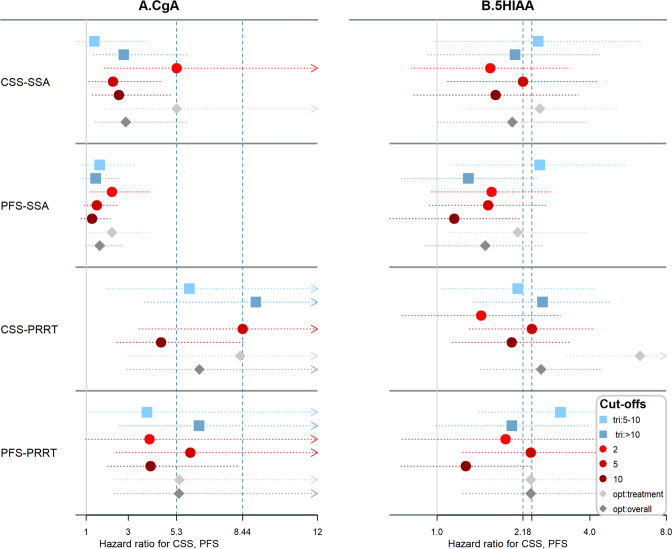
Cancer-specific (CSS) and progression-free survival (PFS) for patients treated with somatostatin analogues (SSA) or peptide receptor radionuclide therapy (PRRT) at various cut-offs: trichotomized (squares), dichotomized (cycles) and at “optimal” estimated cut-offs (diamonds) for the whole cohort (opt: overall) and per treatment given (opt: treatment). **A** Chromogranin A (CgA), **B** 5-hydroxyindoleacetic acid (5HIAA). Dashed lines in place for “standard” cut-offs giving maximum discrimination for CSS. A CgA cut-off of 2 × ULN (upper limit of normal) seems to discriminate best for SSA, whereas 5 × ULN and trichotomization discriminate well for PRRT. Discrimination by 5HIAA is lower irrespective of cut-off examined. In PRRT the “optimal” cut-off is clearly higher. The estimated “overall” optimal cut-offs (dark grey diamond) did not provide better discrimination in any case

We focused on single SSA and PRRT, the two treatments that are most applicable in current clinical practice. For patients treated with SSA, the estimated optimal cut-offs for baseline CgA were 2 × ULN for both CSS and PFS (Table 4). Indeed, CgA dichotomized at 2 × ULN defines a cohort with worse CSS (HR 5.30, 95% CI 1.85–15.21, *p* < 0.01) and PFS (HR 2.22, 95% CI 1.21–4.07, *p* = 0.01). In both cases HRs are clearly higher compared to other cut-offs (Fig. 2). Trichotomizing CgA at 5–10 × ULN did not seem to provide any additional benefit.

The estimated optimal 5HIAA cut-offs were 4 × ULN for CSS and 1 × ULN for PFS. The optimal estimated cut-offs separate two groups with a moderate HR of 2.54 (95% CI 1.25–5.18, *p* = 0.01) for CSS and 2.08 (95% CI 1.11–3.90, *p* = 0.02) for PFS (Table 3). These results, though statistically significant, are less discriminative in comparison to grouping by CgA. A cut-off at 5 × ULN provides a slightly worse discrimination for CSS (HR 2.18, 95% CI 1.10–4.34, *p* = 0.02). Using two cut-off intervals at 5× and 10 × ULN unexpectedly resulted in lower HRs for the >10 × ULN compared to the 5–10 × ULN group.

### Peptide receptor radionuclide therapy (PRRT)

For patients treated with PRRT, the estimated optimal CgA cut-offs were 4 × ULN for CSS and 7 × ULN for PFS. Using two cut-offs at 5× and 10 × ULN clearly distinguished three groups with HR of 5.90 (95% CI 1.97–17.66) and 9.06 (95% CI 3.75–21.88) for CSS, 3.88 (95% CI 1.13–13.29) and 6.36 (95% CI 2.58–15.72) for PFS. However, a single cut-off at 5 × ULN provided similar discriminative value (HR 8.44, 95% CI 3.51–20.31, *p* < 0.01 for CSS and 5.94, 95% CI 2.41–14.63, *p* < 0.01 for PFS), is probably easier to use and provides higher HRs compared to other single cut-offs (Table 2 and Fig. 2).

The estimated optimal cut-offs for 5HIAA were 18× for CSS and 5× for PFS. As is the case with SSA, 5HIAA provided worse discriminative value in comparison with CgA, with similar HRs in the 2–10 × ULN range, which are more frequently used in the literature. The optimal HR in our cohort was provided at higher cut-off values for CSS, and discriminated sufficiently for CSS (HR 6.30, 95% CI 3.24–12.27, *p* < 0.01) and moderately for PFS (HR 2.34, 95% CI 1.26–4.36, *p* = 0.01).

### Changes in biomarkers as prognostic markers of response

#### Somatostatin analogues (SSA)

Biochemical test results at 6 months after treatment start were available for 98 (CgA) and 99 (5HIAA) patients among those who initially had a raised CgA and 5HIAA, respectively. The median reduction of CgA and 5HIAA were 43% and 41% respectively for all patients, 19% and 31% for patients starting treatment with single SSA, and 51% and 53% for patients starting a combination treatment (mostly with IFN and in nine cases with PRRT) at first line. Thirty-nine percent of all patients, 31% of patients treated with single SSA and 53% of patients starting on combined treatment achieved a reduction of CgA at 6 months of at least 50% and were classified as partial responders; the corresponding numbers for 5HIAA were 39%, 31% and 50%.

**Fig. 3:**
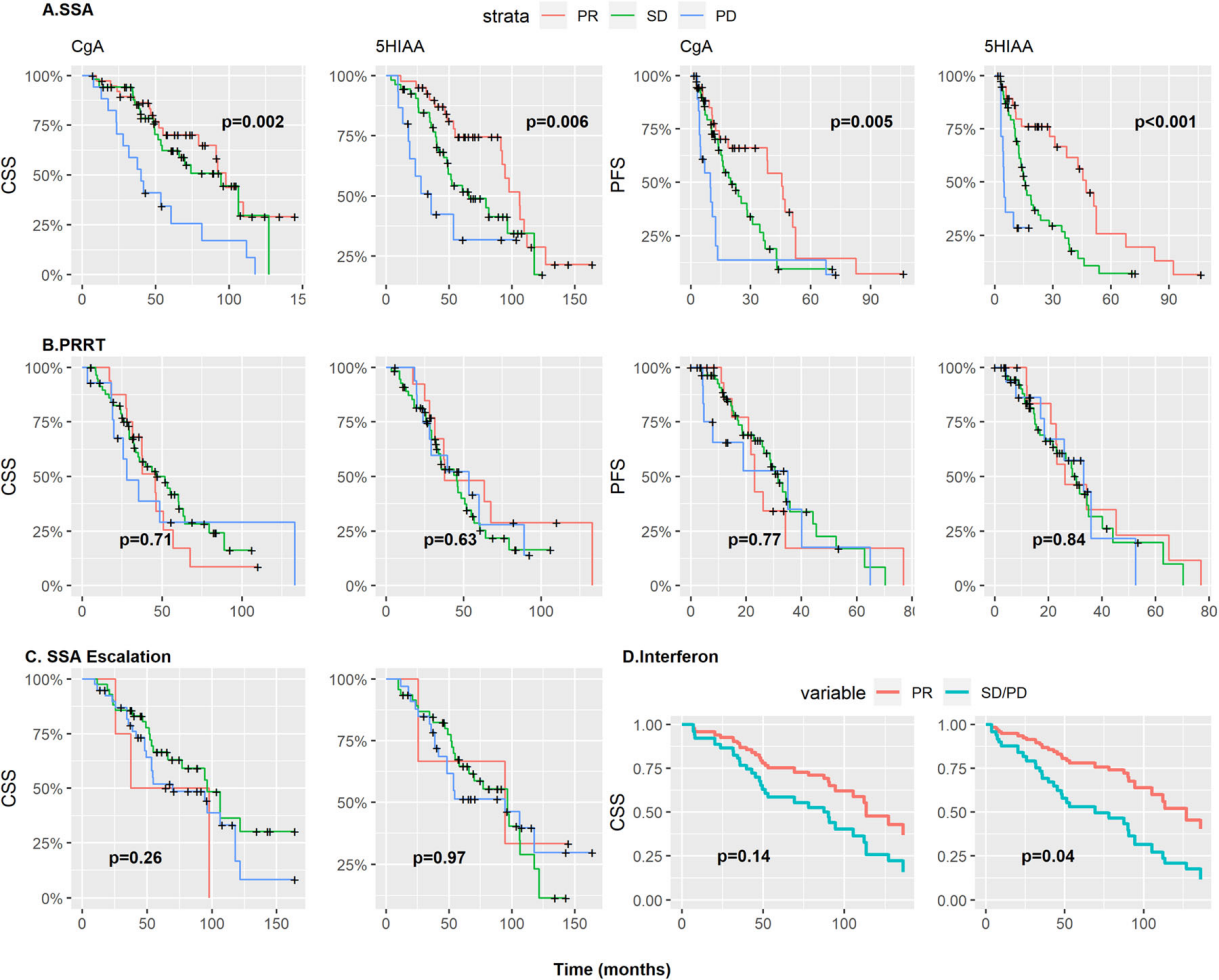
Cancer-specific survival (CSS) and progression-free survival (PFS) after treatment with somatostatin analogues (SSA) (**A**), peptide receptor radionuclide therapy (PRRT) (**B**) and SSA dose escalation (**C**) stratified by 6-month biochemical response. Adjusted survival curves for CSS in patients treated with interferon, as a function of biochemical response for the cox model, stratified for concomitant use of SSA (**D**). CgA Chromogranin A, 5HIAA 5-hydroxyindoleacetic acid, PR partial response, SD stable disease, PD progressive disease. *P* values derived from log-rank test (**A**–**C**) and cox models (**D**)

Six-month reductions of CgA and 5HIAA correlated well with PFS and CSS for patients treated with single SSA, both as continuous and dichotomous variables (Table 5). It is worth noting that although CSS was reduced for patients progressing biochemically within 6 months of SSA start, the difference between patients having SD and those having PR was marginal (Fig. 3).

**Table 5:** Prognostic value of CgA (DCgA) and 5HIAA (D5HIAA) changes at 6 months from treatment start for CSS, PFS

	SSA single				SSA combined				IFN				PRRT				Dose escalation			
	CSS		PFS		CSS		PFS		CSS		PFS		CSS		PFS		CSS		PFS	
	HR [95% CI]	*p*	HR [95% CI]	*p*	HR [95% CI]	*p*	HR [95% CI]	*p*	HR [95% CI]	*p*	HR [95% CI]	*p*	HR [95% CI]	*p*	HR [95% CI]	*p*	HR [95% CI]	*p*	HR [95% CI]	*p*
	*N* = 62		*N* = 62		*N* = 36		*N* = 36		*N* = 25		*N* = 24		*N* = *87*		*N* = *87*		*N* = *85*		*N* = *83*	
DCgA	3.18 [1.82;5.56]	**<0.001**	3.08 [1.86;5.10]	**<0.001**	0.92 [0.41;2.07]	0.842	1.10 [0.55;2.19]	0.782	1.14 [0.59;2.19]	0.699	1.22 [0.62;2.41]	0.563	1.37 [0.94;1.98]	0.099	1.36 [0.99;1.86]	0.058	1.11 [0.99;1.25]	0.078	1.12 [1.03;1.23]	**0.010**
DCgA, Grouped		**<0.001**		**<0.001**		0.855		0.164		0.698	NA			0.810		0.816		0.291		**<0.001**
SD	2.47 [0.79;7.68]		1.78 [0.89;3.55]		1.28 [0.51;3.23]		2.13 [0.95;4.79]		1.68 [0.20;14.1]		NA		0.83 [0.43;1.61]		0.93 [0.44;1.98]		0.46 [0.14;1.58]		0.66 [0.19;2.22]	
PD	7.33 [2.34;22.9]		5.19 [2.21;12.2]		0.94 [0.21;4.30]		1.24 [0.33;4.63]		2.29 [0.27;19.2]		NA		1.00 [0.41;2.45]		1.21 [0.46;3.17]		0.68 [0.20;2.29]		2.83 [0.85;9.44]	
	*N* = 51		*N* = 51		*N* = 38		*N* = 38		*N* = 20		*N* = 20		*N* = *79*		*N* = *79*		*N* = *73*		*N* = *71*	
D5HIAA	1.47 [1.16;1.86]	**0.001**	1.37 [1.11;1.68]	**0.004**	1.05 [0.60;1.84]	0.868	1.49 [0.96;2.30]	0.075	1.89 [1.14;3.12]	**0.013**	1.34 [0.94;1.90]	0.102	1.22 [0.76;1.95]	0.406	1.51 [0.88;2.59]	0.134	1.01 [0.81;1.26]	0.901	1.07 [0.92;1.24]	0.378
D5HIAA, Grouped		**<0.001**		**<0.001**		0.204		0.584		0.246		0.269		0.635		0.764		0.722		**0.001**
SD	2.04 [0.66;6.25]		2.22 [1.00;4.96]		2.06 [0.88;4.85]		1.43 [0.67;3.06]		1.50 [0.17;13.5]		2.08 [0.21;20.1]		1.44 [0.66;3.14]		1.23 [0.57;2.66]		1.18 [0.28;5.05]		1.01 [0.24;4.34]	
PD	10.7 [3.03;37.9]		11.2 [3.64;34.3]		0.83 [0.11;6.55]		1.56 [0.44;5.56]		3.72 [0.43;32.0]		4.62 [0.49;43.3]		1.24 [0.46;3.31]		1.45 [0.53;4.00]		1.48 [0.34;6.46]		3.04 [0.71;13.1]	

Significant values are marked in bold numbers. Hazard ratios (HR) and *p* values calculated with cox models

*DCgA* Delta Chromogranin A, change within 6 months, *D5HIAA* Delta 5-hydroxyindoleacetic acid, change within 6 months, *SSA* somatostatin analogues, *PR* partial response, *PD* progressive disease, *SD* stable disease, *IFN* Interferon-alpha, *PRRT* peptide receptor radionuclide therapy, *CSS* cancer-specific survival, *PFS* progression-free survival, *NA* not available

There were 133 events of escalation of SSA dose in 98 patients. The median change for CgA and 5HIAA was an increase of 17% and 15% respectively after the dose escalation. The biochemical response rate (RR) was 4.7% and 4.1% for CgA and 5HIAA, while 48% and 58%, respectively, had SD at the 6-month control. Changes in CgA as a continuous variable showed a non-statistically significant correlation with CSS (HR 1.11, 95% CI 0.99–1.25, *p* = 0.08). There was no correlation between early 5HIAA changes as a continuous variable and either CSS or PFS. For both CgA and 5HIAA as dichotomous variables, there was no correlation with CSS (Table 5 and Fig. 3).

#### Peptide receptor radionuclide therapy (PRRT)

The median change of CgA and 5HIAA was a reduction of 15% and 12% respectively after PRRT, with RR of 18% and 18% for CgA and 5HIAA, while 66% and 67%, respectively, had stable markers at the 6-month control. Reductions in CgA and in 5HIAA did not correlate with either PFS or CSS (Fig. 3). Results are summarized in Table 5.

Patients treated with PRRT were treated in parallel with various doses of SSA; Thirty-six percent of patients with available data were treated with up to standard doses (30 mg Sandostatin^®^ LAR /4 weeks or 120 mg Somatuline^®^ Autogel/4 weeks), 34% with SSA every 3 weeks and 30% with SSA every 2 weeks or higher doses. We examined whether different SSA doses could potentially mask the early effect of PRRT on CgA and 5HIAA levels stratifying for standard versus higher SSA doses. In this case early reductions of CgA had a borderline significant correlation with CSS (HR 1.45, 95% CI 1.02–2.05, *p* = 0.04), whereas early reductions of 5HIAA did not show any correlation (HR 1.29, 95% CI 0.80–2.07, *p* = 0.29). The correlation between early reduction of CgA and CSS remained significant even after correcting for baseline hormonal values, Ki-67 and performance status (Supplementary Table 3).

#### Interferon-alpha (IFN)

Of the 97 treatment cases with IFN during the study period, 58 had evaluable biomarkers both at baseline and after 6 months of treatment. Of those, 58% initiated treatment concomitantly, or shortly after, the initiation of SSA, as was the clinical routine during the early study period. The median reduction of CgA and 5HIAA was 14% and 31% for the total population, with RR of 29% and 34%. However, at least part of this effect will be due to the SSA analogue given concomitantly: For 35 patients starting treatment with IFN as single treatment, the median CgA increased by 5% and median 5HIAA decreased by 23% at 6 months from treatment start. Still, one out of seven patients had a more than 50% reduction of CgA and/or 5HIAA, and almost two thirds of evaluable patients had stabilization of those biochemical markers.

Because of the small number of patients, we examined patients having PD or SD together and compared them with responders; the groups were stratified by concomitant or not start of IFN. HR for CSS for responders vs. non-responders was statistically significant for 5HIAA (HR 2.59, 95% CI 1.04–6.43, *p* = 0.04) but not for CgA (HR 1.91, 95% CI 0.80–4.56, *p* = 0.14). However only three of 19 evaluable responders were in the IFN single group and this result should be interpreted with caution.

### Changes in biomarkers and early change of treatment

There were 83 cases of early biomarker progression: 23 on SSA, 11 on IFN, 16 on PRRT and 33 after SSA dose escalation. In 31 cases both CgA and 5HIAA increased by >25%, in 25 cases only CgA and in 27 cases only 5HIAA increased. Thirty-nine of those patients changed treatment within a year. In univariate analysis, CSS from treatment start did not differ significantly for those changing treatment early compared to patients who changed treatment more than a year later (HR 1.16, 95% CI 0.67–2.00, *p* = 0.59). However, it is likely that patients changing treatment early had a more aggressive disease and higher baseline hormone levels. In a multivariate analysis for baseline factors associated with more aggressive outcomes (CgA and 5HIAA at baseline, age, Ki-67), HR changed in favour of patients starting escalating their treatments earlier, but this was not statistically significant (HR 0.70, 95% CI 0.37–1.33, *p* = 0.28).

## Discussion

The present study examined the prognostic and predictive ability of two widely used biomarkers in clinical routine, plasma CgA and urine 5HIAA, focusing on their baseline values and relatively early changes during treatment. Unlike most previous studies that included various locations and histopathological grades of NET, it focused on a uniform cohort of metastatic siNET G2 patients. The prognostic value of CgA and 5HIAA for the three major medical treatments used during the study period was examined. It was found that the level of baseline CgA and in certain cases 5HIAA are prognostic of CSS irrespective of treatment modality, and of PFS in patients treated with PRRT and possibly IFN. Changes of CgA and 5HIAA levels at 6 months of treatment correlate well with CSS and PFS in patients treated with SSA, but not in patients treated with PRRT. The number of patients treated with single IFN was too small to draw any definite conclusions.

Elevated baseline CgA levels have long been considered as markers of poor outcome. Indeed, higher CgA levels correlate with high metastatic load [22–24], and with poorer survival in the metastatic setting [22, 24]. Prospective analyses of the RADIANT trials provide evidence of a shorter survival in patients with elevated baseline CgA, using a cut-off of 2 × ULN [12, 15]. The same cut-off was used in a recently published randomized trial comparing interferon with bevacizumab [8]. Other authors have used cut-offs ranging from 1× to 10 × ULN [7, 10, 11, 13, 14], or even studied CgA levels divided into three groups [16, 25]. We examined three different cut-offs used in the literature (2×, 5× and 10 × ULN), as well as “optimal” cut-offs. In our cohort, a standard cut-off of 2 × ULN presents a better discriminative value in patients treated with SSA, whereas 5 × ULN seems more appropriate in combination with PRRT and probably IFN. The estimated “optimal” cut-offs presented only marginal, if any, advantage over those routinely used in the literature.

A recently published prospective study showed only a weak association between changes of CgA and changes in tumour burden [26]. Post hoc analysis from phase 3 trials showed that an early decrease in CgA related to a decreased risk of PD for SSA [18] and everolimus [17] but not for PRRT [14]. A post hoc analysis of the NETTER-1 phase 3 trial comparing PRRT with high-dose SSA showed that there was a statistically significant correlation between 6-month radiological response and PFS for patients treated with SSA but not with PRRT [27]. It is also worth noting that in the case of another well-studied radionuclide used in the treatment of metastatic prostate cancer, Radium-223, changes of the tumour marker prostate-specific antigen is not considered a reliable surrogate marker for survival [28]. These results are largely consistent with our study findings. All trials above used mixed study populations, with less than half of the study population having siNETs. Two recent reviews of CgA as a biomarker reached somewhat contradicting conclusions: Both agree that higher baseline CgA is associated with shorter PFS. When it comes to early response evaluation, the first review indicates that an early response is associated with better clinical outcomes [29] and the second that circulating CgA does not represent a valid marker of morphological evolution of disease and has therefore no utility in this setting [30].

Less is known about the prognostic and predictive value of 5HIAA in siNETs. Zandee et al. reported that baseline 5HIAA at a cut-off of 10 × ULN is a negative prognostic factor of overall survival (OS) in univariate, but not multivariate analysis [25]. On the other hand, Laskaratos et al. reported that 5HIAA at a cut-off of 10 × ULN together with age were the only factors that remain significant in multivariate analysis for OS of siNETs with desmoplasia [16]. Data about 5HIAA changes associated with specific treatments are limited. In our study, 5HIAA seems to be prognostic of PFS and CSS in patients treated with PRRT, but not with single SSA. It is also significant as a prognostic factor for CSS for patients treated with interferon. The optimal cut-off is higher than that for CgA but varies between treatments, and HRs are generally similar in the range of 2–10 × ULN. At least in the case of PRRT the optimal cut-off for CSS might be significantly higher and in our cohort it was 18 × ULN.

We examined early biomarker changes in some specific situations: First we showed that in a group of patients treated with escalated doses of SSA, early continuous decreases of CgA, but not 5HIAA, had borderline correlation with CSS and PFS. Moreover, we hypothesized that early biomarker changes in patients treated with PRRT might be masked by concomitant treatment with SSA. Unfortunately, we could not examine biomarker changes in an SSA-naive population, but we examined differences depending on the different baseline SSA doses. Early changes of CgA but not 5HIAA showed a borderline correlation with CSS after correcting for baseline SSA doses.

As more treatment options become available, it is important to investigate if early intensification of treatment can improve survival. Approximately half of the patients with early biochemical progression changed treatment within a year of treatment start. We compared patients with early treatment changes with those who remained on the same treatment for a longer time. In univariate analysis there was no difference in CSS (HR 1.16, 95% CI: 0.67–2.00, *p* = 0.59), but the HR switched in favour of early treatment changes when adjusting for known prognostic factors of poor response in a multivariate analysis (HR 0.70, 95% CI 0.37–1.33, *p* = 0.28). A larger study could give a definitive answer to this question.

Monoanalytes, such as CgA and 5HIAA have been criticized for lacking specificity and sensitivity [31, 32]. Genomic assays such as the NETest^®^ have been argued to provide a more precise alternative [32]. However, trials to date have examined the prognostic ability of the NETest^®^ in relation to disease status and progression instead of OS. Besides, genomic tests have struggled to become mainstream in most oncologic fields and are almost never used in the metastatic setting: For example the 15-year-old Oncotype DX^®^ in breast cancer still had 35% adoption rate among US physicians and as low as <20% in some areas in Europe [33, 34]. Simple monoanalytes are still used routinely in several solid tumours and, despite their limitations, are probably unlikely to be completely replaced by multi-genomic assays in the very near future.

The study has several limitations. First, the study population is mostly that of a tertiary referral centre, and might not be representative of the general population. Second, this is a retrospective study with laboratory tests spanning over a 20-year period and there is considerable variation between different methods of measuring CgA and 5HIAA. However, more than two-thirds of tests in SSA and IFN patients and virtually all tests in PRRT patients were conducted in a single reference laboratory. Finally, treatment patterns changed during the study period; most notably IFN concomitantly with SSA as a first line treatment is rarely used nowadays, and the IFN results might not be applicable to patients treated with IFN at second or later lines.

In conclusion, we have shown that CgA and to a lesser extent 5HIAA baseline levels are associated with CSS in patients with siNETs G2, irrespective of treatment used, and with PFS in patients treated with PRRT and we suggest optimal cut-off points for dichotomizing those variables. The reductions of CgA and 5HIAA at 6 months from treatment start have prognostic utility in patients treated with SSA, but not in patients treated with PRRT.

## Supplementary information

Supplementary Table 1

Supplementary Table 2

Supplementary Table 3
