# Supplementary material for: Assessment of hormonal levels as prognostic markers and of their optimal cut-offs in small intestinal neuroendocrine tumours grade 2
Source: Endocrine. 2020 Nov 26;72(3):893–904. doi: 10.1007/s12020-020-02534-8 (PMC8159831; doi:10.1007/s12020-020-02534-8)
Supplement: Supplementary file 1 — Supplementary Table 1 [file 12020_2020_2534_MOESM1_ESM.docx]

**Supplementary table 1. Baseline CgA and 5HIAA as predictors for CSS (all treatments combined)**

|  | CSS (N=241) | | | |
| --- | --- | --- | --- | --- |
|  | **CgA** | | **5HIAA** | |
|  | **HR [95% CI]** | **p** | **HR [95% CI]** | **p** |
| Continuous | 1.006 [1.005;1.008] | **<0.01** | 1.01 [1.01;1.02] | **<0.01** |
| 5-10x |  | **<0.01** |  | **<0.01** |
| 5-10x | 2.06 [1.15;3.67] |  | 1.69 [1.06;2.70] |  |
| >10 | 4.03 [2.67;6.07] |  | 2.38 [1.64;3.48] |  |
| >2 | 3.66 [2.06;6.50] | **<0.01** | 1.56 [1.05;2.31] | **0.03** |
| >5 | 3.43 [2.30;5.10] | **<0.01** | 2.10 [1.49;2.96] | **<0.01** |
| >10 | 3.30 [2.31;4.72] | **<0.01** | 2.09 [1.47;2.97] | **<0.01** |
| >Optimal | 3.56 [2.43;5.22] | **<0.01** | 2.17 [1.55;3.05] | **<0.01** |

Prognostic value of baseline Chromogranin A (CgA) and of 5-hydroxyindoleacetic acid (5HIAA) for cancer-specific (CSS) given as continuous values, at “standard” cut-offs 2x, 5x, 10xULN and at “optimal” estimated cut-offs for the whole cohort. Significant p-values are marked in bold numbers
