# Supplementary material for: Assessment of hormonal levels as prognostic markers and of their optimal cut-offs in small intestinal neuroendocrine tumours grade 2
Source: Endocrine. 2020 Nov 26;72(3):893–904. doi: 10.1007/s12020-020-02534-8 (PMC8159831; doi:10.1007/s12020-020-02534-8)
Supplement: Supplementary file 2 — Supplementary Table 2 [file 12020_2020_2534_MOESM2_ESM.docx]

**Supplementary table 2. Prognostic value of CgA (DCgA) and 5HIAA (D5HIAA) changes at 6 months from treatment start for CSS, PFS**

|  | **Sensitivity** | **Specificity** | **PPV** | **NPV** | **Sensitivity** | **Specificity** | **PPV** | **NPV** | **Sensitivity** | **Specificity** | **PPV** | **NPV** | **Sensitivity** | **Specificity** | **PPV** | **NPV** |
| --- | --- | --- | --- | --- | --- | --- | --- | --- | --- | --- | --- | --- | --- | --- | --- | --- |
|  | **CgA** | | | | | | | | | | | | | | | |
|  | **CgA** | | | | | | | | | | | | | | | |
|  | **CSS at 3 years** | | | | | | | | | | | | | | | |
| **Cut-off** | **SSA single** | | | | **SSA combined** | | | | **IFN** | | | | **PRRT** | | | |
| **2** | 1 | 0.36 | 0.33 | 1 | 0.87 | 0.25 | 0.20 | 0.90 | 1 | 0.35 | 0.43 | 1 | 1 | 0.15 | 0.48 | 1 |
| **5** | 0.82 | 0.52 | 0.35 | 0.9 | 0.87 | 0.47 | 0.26 | 0.94 | 0.88 | 0.71 | 0.59 | 0.92 | 0.97 | 0.49 | 0.60 | 0.95 |
| **10** | 0.64 | 0.68 | 0.39 | 0.86 | 0.87 | 0.56 | 0.30 | 0.95 | 0.77 | 0.82 | 0.68 | 0.88 | 0.91 | 0.69 | 0.70 | 0.91 |
| **Optimal: treatment** | 1 | 0.36 | 0.33 | 1 | 0.62 | 0.89 | 0.55 | 0.92 | 0.66 | 1 | 1 | 0.86 | 0.97 | 0.44 | 0.57 | 0.95 |
|  |  |  |  |  |  |  |  |  |  |  |  |  |  |  |  |  |
| **Optimal** | 0.76 | 0.64 | 0.4 | 0.89 | 0.87 | 0.5 | 0.27 | 0.95 | 0.88 | 0.71 | 0.59 | 0.92 | 0.94 | 0.51 | 0.60 | 0.91 |
|  | **PFS at 1 year** | | | | | | | | | | | | | | | |
| **2** | 0.94 | 0.45 | 0.57 | 0.90 | 1 | 0.25 | 0.20 | 1 | 1 | 0.36 | 0.50 | 1 | 1 | 0.10 | 0.19 | 1 |
| **5** | 0.72 | 0.53 | 0.55 | 0.70 | 1 | 0.44 | 0.25 | 1 | 0.90 | 0.73 | 0.68 | 0.92 | 1 | 0.31 | 0.24 | 1 |
| **10** | 0.50 | 0.66 | 0.54 | 0.62 | 1 | 0.53 | 0.29 | 1 | 0.69 | 0.73 | 0.62 | 0.78 | 0.92 | 0.44 | 0.26 | 0.96 |
| **Optimal: treatment** | 0.94 | 0.45 | 0.57 | 0.90 | 0.86 | 0.81 | 0.46 | 0.97 | 0.90 | 0.73 | 0.68 | 0.92 | 1 | 0.36 | 0.25 | 1 |
|  |  |  |  |  |  |  |  |  |  |  |  |  |  |  |  |  |
| **Optimal** | 0.66 | 0.63 | 0.59 | 0.70 | 1 | 0.47 | 0.26 | 1 | 0.90 | 0.73 | 0.68 | 0.92 | 1 | 0.34 | 0.24 | 1 |
|  | **5HIAA** | | | | | | | | | | | | | | | |
|  | **CSS at 3 years** | | | | | | | | | | | | | | | |
| **2** | 0.68 | 0.41 | 0.29 | 0.78 | 1 | 0.31 | 0.26 | 1 | 0.74 | 0.47 | 0.39 | 0.8 | 0.89 | 0.26 | 0.49 | 0.76 |
| **5** | 0.47 | 0.67 | 0.34 | 0.78 | 0.78 | 0.53 | 0.28 | 0.91 | 0.50 | 0.71 | 0.43 | 0.76 | 0.69 | 0.53 | 0.54 | 0.68 |
| **10** | 0.31 | 0.78 | 0.33 | 0.76 | 0.78 | 0.69 | 0.38 | 0.93 | 0.37 | 0.88 | 0.59 | 0.76 | 0.47 | 0.76 | 0.61 | 0.64 |
| **Optimal: treatment** | 0.62 | 0.57 | 0.34 | 0.81 | 0.56 | 0.83 | 0.44 | 0.89 | 0.50 | 0.88 | 0.65 | 0.80 | 0.44 | 0.97 | 0.93 | 0.68 |
|  |  |  |  |  |  |  |  |  |  |  |  |  |  |  |  |  |
| **Optimal** | 0.42 | 0.67 | 0.31 | 0.76 | 0.78 | 0.56 | 0.3 | 0.91 | 0.50 | 0.82 | 0.56 | 0.78 | 0.66 | 0.58 | 0.56 | 0.68 |
|  | **PFS at 1 year** | | | | | | | | | | | | | | | |
| **2** | 0.67 | 0.46 | 0.50 | 0.63 | 1 | 0.24 | 0.20 | 1 | 0.76 | 0.45 | 0.45 | 0.77 | 0.92 | 0.20 | 0.20 | 0.92 |
| **5** | 0.45 | 0.69 | 0.54 | 0.61 | 0.86 | 0.49 | 0.24 | 0.95 | 0.56 | 0.73 | 0.54 | 0.74 | 0.86 | 0.45 | 0.25 | 0.94 |
| **10** | 0.27 | 0.74 | 0.46 | 0.56 | 0.86 | 0.65 | 0.31 | 0.96 | 0.33 | 0.82 | 0.52 | 0.68 | 0.42 | 0.65 | 0.21 | 0.84 |
| **Optimal: treatment** | 0.85 | 0.36 | 0.52 | 0.74 | 0.86 | 0.51 | 0.25 | 0.95 | 0.66 | 0.55 | 0.46 | 0.73 | 0.86 | 0.45 | 0.25 | 0.94 |
|  |  |  |  |  |  |  |  |  |  |  |  |  |  |  |  |  |
| **Optimal** | 0.45 | 0.69 | 0.54 | 0.61 | 0.86 | 0.49 | 0.24 | 0.95 | 0.56 | 0.73 | 0.54 | 0.74 | 0.86 | 0.45 | 0.25 | 0.94 |

Sensitivity, specificity, positive predictive value (PPV) and negative predictive value (NPV) for cancer-specific (CSS) and progression-free survival (PFS) given as continuous values, at cut-offs 2x, 5x, 10xULN and at “optimal” estimated cut-offs for the whole cohort (optimal) and per treatment given (optimal: treatment). CgA: Chromogranin A, 5HIAA: 5-hydroxyindoleacetic acid, DCgA: Delta Chromogranin A, change within 6 months, D5HIAA: Delta 5-hydroxyindoleacetic acid, change within 6 months, SSA: Somatostatin analogues, IFN: Interferon-alpha, PRRT: peptide receptor radionuclide therapy, CSS: cancer-specific survival, PFS: progression-free survival, PPV: positive predictive value, NPV: negative predictive value
