# Supplementary material for: Assessment of hormonal levels as prognostic markers and of their optimal cut-offs in small intestinal neuroendocrine tumours grade 2
Source: Endocrine. 2020 Nov 26;72(3):893–904. doi: 10.1007/s12020-020-02534-8 (PMC8159831; doi:10.1007/s12020-020-02534-8)
Supplement: Supplementary file 3 — Supplementary Table 3 [file 12020_2020_2534_MOESM3_ESM.docx]

**Supplementary table 3**. Multivariate analysis stratified for somatostatin analogue dose at initiation of PRRT

|  |  | | | | | |
| --- | --- | --- | --- | --- | --- | --- |
|  | **Dependent variable: CSS** | | | |  |  |
|  |  | | | |  |  |
|  | **HR** |  |  | **p** |  |  |
| Ki-67> 5% | 1.923 |  |  | 0.107 |  |  |
|  | [1.128; 2.719] | | |  |  |  |
| Performance status ≥1 | 1.667 |  |  | 0.190 |  |  |
|  | [0.903; 2.432] | | |  |  |  |
| Baseline CgA | 1.024 | | | **<0.001** |  |  |
|  | [1.013; 1.035] | | |  |  |  |
| Baseline 5HIAA | 1.008 |  |  | 0.729 |  |  |
|  | [0.963; 1.053] | | |  |  |  |
| DCgA | 1.612 | |  | **0.024** |  |  |
|  | [1.198; 2.027] | | | |  |  |
| Observations | 73 |  |  |  |  |  |

Multivariate analysis of cancer-specific survival (CSS) in relation to Ki-67, performance status, baseline Chromogranin A (CgA) and 5-hydroxyindoleacetic acid (5HIAA) and early changes of CgA (DCgA, Delta Chromogranin A, change within 6 months), stratified by somatostatin analogue dose at peptide receptor radionuclide therapy (PRRT) start. Significant values are marked in bold numbers
